# Comparative pharmacology of oral fluoropyrimidines: a focus on pharmacokinetics, pharmacodynamics and pharmacomodulation

**DOI:** 10.1038/sj.bjc.6601973

**Published:** 2004-07-27

**Authors:** G Milano, J-M Ferrero, E François

**Affiliations:** 1 Oncopharmocology Unit, Centre Antoine-Lacassagne, 33, Avenue de Valombrose, 06189 Nice Cedex 2, France

**Keywords:** oral chemotherapy, UFT, capecitabine

## Abstract

The main purpose of the present review article was to shed light on the different 5-fluorouracil (5-FU) prodrugs by underlining their respective pharmacological features in terms of metabolic activation, dihydropyrimidine dehydrogenase inhibition, pharmacokinetic profile and biomodulation ability. Oral fluoropyrimidines differ particularly as concerns their pharmacokinetic profile and especially in the delivery of circulating 5-FU. More clinical studies need to be performed incorporating tumour predictive markers during oral fluoropyrimidine-based treatment. The new possibilities are to achieve pharmacomodulation of oral fluoropyrimidines, notably for UFT and capecitabine, that open up the prospect of establishing significant novel treatment protocols based on drug combinations.

5-Fluorouracil (5-FU) remains one of the most commonly prescribed anticancer drugs with significant activity against cancers of the gastrointestinal tract, head and neck and breast. Repeated intravenous administrations or protracted infusions are heavy for patients. Other oral 5-FU prodrugs, with new pharmacological characteristics, are now emerging in the clinical area in oncology (Diasio, 1999). These 5-FU prodrugs differ markedly in their mode of activation, their pharmacokinetic behaviour, particularly in terms of dihydropyrimidine dehydrogenase (DPD) inhibition, and their pharmacologic modulation. Table 1

**Table 1:** Oral fluoropyrimidines under clinical evaluation

**Compound**	**Chemical name**	**5-FU prodrug**	**Effect on DPD**
Eniluracil (Glaxo-Wellcome) (Clinical development currently stopped)	5-Ethynyluracil	No	Inactivator (complete DPD inhibition)
UFT (Orzel®, Bristol-Myers Squibb, contains UFT plus leucovorin)	Uracil+tegafur	Yes	Inhibitor
S-1 (Bristol-Myers Squibb)	5-Chloro-2, 4 dihydropyrimidine+tegafur+potassium oxonate	Yes	Inhibitor
BOF-A2 (Emitefur®, Otsuka America Pharmaceutical)	1-Ethoxymethyl-5-fluorouracil+3-cyano-2,6 dihydropyrimidine	Yes	Inhibitor
Capecitabine (Xeloda®, Roche)	N^4^-pentyloxycarbonyl-5'-deoxy-5-fluorocytidine	Yes	No DPD inhibitor

describes the 5-FU prodrugs currently at more or less advanced stage of clinical development. Previous reviews on oral fluoropyrimidines have provided extensive description of the different characteristics of these drugs (Diasio, 1999). The purpose of the present review is to propose a transversal pharmacological analysis of these oral fluoropyrimidines. Thus, special attention will be paid to metabolic activation, DPD inhibition, pharmacokinetic profile and biomodulation ability leading to compare oral fluoropyrimidines between them at the light of these latter pharmacological criteria. This information could promote a better understanding of their pharmacological properties as well as enabling optimal clinical use in the framework of the currently approved therapeutic schedules and in the context of future clinical developments.

## DPD INHIBITION

More than 80% of an administered dose of 5-FU is eliminated by catabolism through DPD which is the rate-limiting enzyme of pyrimidine catabolism (Grem, 1996). The combination of DPD inhibitors with 5-FU itself or 5-FU prodrugs has several potential pharmacologic benefits conferred by the inhibition of 5-FU catabolism during gastrointestinal absorption and first pass in the liver. Furthermore, DPD inhibition improves pharmacokinetic behaviour of delivered 5-FU by reducing interpatient variability and by increasing 5-FU half-life. This latter benefit is particularly useful in limiting repeated oral administrations of the drug which is uncomfortable for patients. A large spectrum of DPD inhibitors are used in combination with 5-FU or 5-FU prodrugs. At one extreme, there is eniluracil, which has no cytotoxic activity but which constitutes the most efficacious DPD inhibitor, acting by DPD inactivation through the formation of a covalent bond; this covalent reaction irreversibly inhibits DPD activity at its active site. At the other extreme is UFT including uracil (U) which is a simple competitive inhibitor of DPD activity since it is the natural substrate of DPD. S-1 incorporates another DPD competitor, 5-chloro-2,4-dihydroxypyridine which is 200-fold more potent than U (Shimasaka *et al*, 1996). BOF-A2 is an oral prodrug of 5-FU and 3-cyano-2,6-dihydropyrimidine (CNDP). BOF-A2 is rapidly transformed by esterification into two 1 : 1 components, namely, 1-ethoxymethyl-5-fluorouracil and CNDP. CNDP is also a competitive inhibitor of DPD with a potency of 2000 times greater than that of U (Tatsumi *et al*, 1993). Capecitabine does not incorporate a DPD inhibitor and is converted to the cytotoxic moiety 5-FU in target tissue through a series of three metabolic steps (Diasio, 1999). The development of the compound RO0094889 which generates the DPD inhibitor 5-vinyluracil preferentially in tumours by the same enzymes as in capecitabine activation (Hattori *et al*, 2003) may lead to an interesting combination RO0094889–capecitabine to be further explored at the clinical level (Endo *et al*, 2003).

## PHARMACOKINETICS

Oral fluoropyrimidines differ particularly as concerns their pharmacokinetic profile and especially in the delivery of circulating 5-FU. The DPD inactivator eniluracil is administered with 5-FU in a 10 : 1 ratio and produces 5-FU directly in the blood compartment. 5-FU pharmacokinetics during multiple oral dosing of eniluracil and 5-FU have been reported by Baker *et al* (2000). Elimination half-life of 5-FU was around 4.0 h. Between day 2 and day 29 the main pharmacokinetic parameters remained constant, notably for the values of the total body clearance (Baker *et al*, 2000). Interestingly, Adjei *et al* (2002) recently reported on a randomised, open-label, cross-over study comparing continuous venous infusion (CVI) of 5-FU to 5-FU/eniluracil combination. The authors noted that individual 5-FU concentrations during CVI were highly variable whereas those after 5-FU/eniluracil were more reproducible, this lesser variability in 5-FU concentrations following 5-FU/eniluracil being attributable to the inhibition of DPD.

The pharmacokinetics of 5-FU following the administration of S-1 at a standard dose of 80 mg m^−2^ day^−1^ were first reported by Hirata *et al* (1999). This relative stability in the pharmacokinetics of 5-FU during S-1 treatment was not in agreement with the recent data by Peters *et al* (2003), who reported an increase in 5-FU and uracil plasma concentration during repeated daily administration of S-1. Thus, more data are needed about the time-stability of the pharmacokinetics of S-1 during repeated administration. Elimination half-life of 5-FU during S-1 treatment was reported to be in the range of 1.9–2.9 h (Hirata *et al*, 1999). This slowed 5-FU elimination as compared to 5-FU given alone reflects the presence of the DPD inhibitor in S-1. The fate of 5-FU following the administration of BOF-A2 was examined during a phase I dose-escalating trial (200 mg m^−2^ day^−1^ b.i.d. to 300 mg m^−2^ day^−1^ t.i.d., Nemunaitis *et al*, 2000). The mean steady-state concentration of plasma 5-FU was in the range of 30–100 ng ml^−1^. A lack of variation of 5-FU through levels within a day at steady state could be explained by the suppression of circadian variations in 5-FU concentrations due to the DPD inhibition (Petit *et al*, 1988).

Average plasma concentrations of 5-FU generated from FT in patients treated with oral UFT were comparable to those observed in CVI-treated patients (Ho *et al*, 1998). Owing to the presence of uracil the 5-FU elimination half-life was markedly higher during UFT treatment (7.2±3.9 h on day 5) as compared to CVI (0.19 h). Muggia *et al* (1996) examined 5-FU AUC levels as a function of the timed dose of UFT, 300 mg m^−2^ (0800 *vs* 1800 h). Although not significant, higher 5-FU blood exposures (AUC) were observed in the afternoon dose as compared to the morning. The fact that patients were not their own controls limits the conclusions of the latter study but, from a practical point of view, it would be particularly important to know the magnitude of the difference in 5-FU availability between morning and afternoon administration of UFT. A study was undertaken to address the possible pharmacokinetic influence of concurrent oral administration of UFT and LV (Meropol *et al*, 1999). When LV was coadministered with UFT, there was no significant impact on the fates of FT, uracil and, particularly, on 5-FU AUC values. Urien *et al* (2003) recently examined intrapatient variations in the UFT pharmacokinetics on the first cycle of treatment. A steady state was attained for FT and 5-FU at least on day 8 and there was no further cumulative increase in the AUC of these compounds after 1 week of treatment. In contrast, Ho *et al* (2000) noted that repeated treatment with UFT led to more or less marked cumulative increase in most of the clinically relevant pharmacokinetic parameters. Thus, larger clinical pharmacokinetic studies are still needed to examine more thoroughly the evolution of 5-FU concentration profile during repeated UFT treatment. It would be of potential interest to check whether the 7-day rest period after the 28-day treatment course allows or not 5-FU AUC to return to values close to those observed at the start of treatment.

The clinical pharmacokinetics of capecitabine have been recently reviewed by Reigner *et al* (2001). The preferential delivery of 5-FU into the tissues through the intermediary of thymidine phosphorylase (TP) is responsible for its much lower presence (approximately 10 times lower) in plasma than its prodrugs capecitabine, 5′DFCR, 5′DFUR or its catabolites FUH2 and FBAL (Reigner *et al*, 2001). Reigner *et al* (2001) noted that the AUC of capecitabine, 5′-DFCR and 5′-DFUR did not accumulate in plasma after long-term administration. We recently conducted a phase I and pharmacokinetic study of the association of capecitabine–cisplatin in head and neck cancer patients (Pivot *et al*, 2003) and found that there was a significant but moderate increase in postdose (capecitabine, 1000 mg m^−2^) AUC values for 5′DFUR and 5-FU between day 2 and day 15. Interestingly, these pharmacokinetic changes were reversible after the intercycle resting period. However, more knowledge is still needed about the stability of the pharmacokinetic parameters of capecitabine and UFT during their repeated administration.

Perhaps more relevant than plasma pharmacokinetics are data regarding the fate of anticancer drugs in tumours. In colorectal patients treated by UFT, Sadahiro *et al* (2001) examined the respective concentrations of 5-FU in serum, tumour and normal mucosa at various intervals after the final dose of UFT. While the serum 5-FU concentration decreased to very low levels by 24 h following the UFT dose, the intratumour concentration of 5-FU had been lowered to only about half, and drug levels in normal mucosa were maintained at least 48 h after the final dose. Concentrations of 5-FU in the normal mucosa were approximately one-third of those measured in the tumour. Similar observations had been previously reported in head and neck tumours for patients pretreated for 1 week with UFT before surgery (Tachibana *et al*, 1987). Higher concentrations of 5-FU in tumours were reported for colorectal cancer patients treated by capecitabine (Schüller *et al*, 2000). Patients received 1 225 mg m^−2^ twice daily for 5–7 days prior to surgery. The ratio of 5-FU concentrations in tumour to adjacent healthy tissue averaged 3.2 while the mean tissue/plasma 5-FU concentration ratio exceeded 20 for colorectal tumour and ranged from 8 to 10 for other tissues. Data in breast cancer patients treated by capecitabine are more contrasted with no significant differences for the levels of capecitabine and its metabolites between healthy and malignant tissue (Mader *et al*, 2003).

The influence of various covariables including gender, biological functions, food intake and coadministration of other drugs has been examined for the pharmacokinetics of oral fluoropyrimidines. Pharmacokinetic changes due to food have been particularly well studied for UFT and capecitabine. For this latter drug it was shown by Reigner *et al* (1998) that food had marked effects on the AUC of capecitabine only (50% reduction) while the impact on metabolites in plasma was minor. Consequently, it was recommended that capecitabine be administered with food. In contrast it was found for UFT that food significantly decreased the maximal plasma concentrations and AUC values of uracil and 5-FU (Damle *et al*, 2001). These observations led to the conclusion not to administer UFT simultaneously with food. The administration of food with oral 5-FU and eniluracil has also been considered (Shepard *et al*, 2002); the pharmacokinetic data show a slowed absorption of 5-FU and decreased 5-FU *C*_max_ but without significant effect on AUC.

The impact of gastrectomy has not been sufficiently explored for oral fluoropyrimidines. Hirata *et al* (1999) compared the pharmacokinetic parameters of 5-FU following administration of S-1 among patients who underwent gastrectomy and those who did not. They found that the influence of gastrectomy was minor. Few studies have considered the possible influence of deteriorated renal or hepatic functions on the pharmacokinetics of oral fluoropyrimidines and more investigations at this level must be encouraged. This lack of information is surprising as hepatic dysfunction is relatively common in patients with cancer of the breast, colon or rectum, which are tumoural localisations with a high incidence of liver metastases and particularly concerned by the current development of oral fluoropyrimidines. Twelves *et al* (1999) examined the impact of hepatic dysfunction due to liver metastases on the pharmacokinetics of capecitabine and its metabolites. Counterintuitively, maximal plasma concentrations and AUC values of the main metabolites of capecitabine were increased in patients with liver dysfunction. These differences, however, did not reach statistical significance and the authors concluded that in patients with mild-to-moderately impaired hepatic function there is no need to adjust the dose. Cassidy *et al* (1999) applied univariate and multivariate regression analyses to study the influence of age, gender, body surface area and creatinine clearance on the main pharmacokinetic parameters of capecitabine and observed no clinically significant effects, the only statistically significant results being a higher AUC of intact capecitabine in females as compared to males (87% greater in females than in males). However, in patients with renal dysfunction and treated by capecitabine there was a significant increase in 5′DFUR (Poole *et al*, 2002); this led to specifically recommend a dose modification of capecitabine for patients with very low creatinine clearance. More studies are needed to examine the possible influence of altered renal or hepatic function on the pharmacokinetics of oral fluoropyrimidines.

## PHARMACOKINETICS–PHARMACODYNAMICS RELATIONSHIPS

For most oral fluoropyrimidines developed so far, PK–PD relationships were observed most often during phase I trials. For instance, Van Groeningen *et al* (2000) noted, for S-1, that among 5-FU plasma concentrations measured during the 28 days of oral treatment, only the level just before drug intake in week 2 was significantly related to grade 3-4 diarrhoea. A confirmation of this interesting observation during phase II–III trials would be useful in order to identify a 5-FU threshold concentration at risk for toxicity with a possibility for dose adjustment during the treatment course. Steady-state plasma 5-FU concentrations were monitored during a phase I trial combining eniluracil-5FU treatment with radiotherapy (Humerickhouse *et al*, 1999). The authors noted that average drug concentrations correlated inversely with absolute neutrophil count nadirs but no correlation was observed with other toxicities. Muggia *et al* (1996), during the UFT timed-dose study, reported on marked toxicities (including life-threatening haematological toxicities) in patients exhibiting peak levels of 5-FU above 1 *μ*g ml^−1^. Ho *et al* (2000) observed significant correlations between maximal plasma concentration or AUC 0–6 h of 5-FU and the occurrence of diarrhoea or nausea and vomiting during a phase I trial on UFT. This information could be potentially useful in order to perform an early pharmacokinetic-based dose adaptation of UFT. Since capecitabine generates 5-FU directly at cellular level, it can be anticipated that the variability in plasma pharmacokinetics of capecitabine and metabolite will be of little concern for pharmacodynamics. This opinion has been confirmed by clinical pharmacokinetic investigations (Reigner *et al*, 2001).

## PREDICTIVE MARKERS

Whatever the oral fluoropyrimidine considered, all release 5-FU which is the final cytotoxic prodrug. Consequently, our clinical knowledge of the predictive markers for 5-FU-based treatment efficacy should apply to oral fluoropyrimidines. Ichikawa *et al* (2003) examined the relative tumoural expression of DPD and TS for advanced colorectal patients to be treated by UFT. The authors noted that no responding tumours had both high DPD and high TS and the response rate reached a satisfactory 75% in tumours with both low DPD and low TS. Capecitabine ultimately delivers 5-FU at the cellular level through the action of TP; in theory, the flux of 5-FU production can be counterbalanced by a more or less marked opposite flux of 5-FU intracellular catabolism mediated by DPD. This view was recently confirmed by Tsukamoto *et al* (2001), who measured, *in vitro*, the enzyme kinetic parameters of each of the enzymes involved in the activation of capecitabine to 5-FU and elimination. The authors constructed a physiologically based pharmacokinetic model which revealed that the most important factors determining the selective production of 5-FU in tumour tissue after capecitabine administration were activation by TP, nonlinear elimination of 5-FU by DPD and the tumour blood flow rate. These data are in agreement with those reported by Ishikawa *et al* (1998) showing that the efficacy of capecitabine on a large panel of 24 human cancer xenografts was significantly correlated to the tumour TP/DPD ratio. Clinical confirmation of these experimental data would be of great interest. Overall, our current understanding of predictive markers for oral fluoropyrimidine treatment is limited and, with the exception of UFT, remains based on extrapolations from 5-FU clinical studies.

## PHARMACOMODULATION

The first approach for pharmacologically modulating oral fluoropyrimidines is to incorporate a DPD inhibitor. This strategy allows 5-FU to be correctly absorbed, as is the case with the combination eniluracil-5FU (Hirata *et al*, 1999; Adjei *et al*, 2002). DPD inhibition also markedly prolongs 5-FU elimination half-life thus making the oral administration of fluoropyrimidines pharmacokinetically acceptable on the basis of two or three daily intakes. The second approach for a pharmacomodulation of oral fluoropyrimidines involves combination with leucovorin (LV). There is, however, an open debate about the clinical/pharmacological efficacy of LV supplementation during prolonged administration of 5-FU. Solutions to this dilemma are provided by experimental and clinical data. For instance, an interesting study by Codacci-Pisanelli *et al* (1995) examined the effects of adding LV on human cancer-xenografts in mice subcutaneously implanted with pellets releasing 5-FU over a period of 3 weeks. The authors clearly demonstrated that coadministration of LV did not enhance the therapeutic results. On the clinical side, a meta-analysis was previously reported showing the advantage of 5-FU plus LV over 5-FU in terms of objective response rate (Advanced Colorectal Cancer Meta-analysis Project, 1992). However, all reports included in the analysis concerned clinical trials with rapid intravenous 5-FU administrations. A clinical pharmacological study was specifically designed in gastric cancer patients to determine whether orally administered LV enhanced TS inhibition when added to UFT (Ichikura *et al*, 1996). The results showed that TS inhibition rate in tumours was significantly higher in the UFT–LV group than in the UFT group alone. However, the data were obtained following a short period of 3 consecutive days of treatment prior to surgery. Thus, it is not clear, as is the case with the current clinical protocols, whether prolonged exposure to UFT results in an equivalent TS inhibition rate with or without LV supplementation. On the other hand, Van Cutsem *et al* (2000) undertook a randomised phase II trial in advanced colorectal cancer patients aimed at selecting the most appropriate capecitabine regimen for testing in phase III studies. Two arms (2 weeks on/1 week off) differed by the presence or not of daily oral LV. Tumour responses were comparable between the two arms but more toxicity was seen in the LV–capecitabine arm. Thus, we do not dispose of strong experimental and clinical arguments to support LV supplementation during continuous treatment by oral fluoropyrimidines.

Apart from DPD and LV handling which can apply to most oral fluoropyrimidines, there are more specifically product-oriented strategies which can be of potential interest for the pharmacomodulation of these drugs. This is particularly true for UFT and capecitabine. Reports have been published of attempts to modulate the activity of 5′DFUR, and thus that of capecitabine, through the upregulation of TP. Cytotoxic agents, particularly taxanes and irradiation, are able to increase TP activity and result in synergistic interaction with capecitabine on experimental models *in vitro* and *in vivo* (Sawada *et al*, 1998, 1999). However, the exact mechanistic cause of the TP modulation by the cytotoxics has not been clearly elucidated and indirect effects via the upregulation of TNF*α* have been advocated (Sawada *et al*, 1998, 1999). The preclinical findings of supra-additive antitumour effects of combining taxanes and capecitabine have been followed by promising clinical results in breast cancer treatment with a protocol based on a capecitabine–docetaxel combination (O'Shaughnessy *et al*, 2002). Recent findings suggest that cytochrome *P*-4502A6 is the major enzyme responsible for the bioactivation process of ftorafur (Ikeda *et al*, 2000). Further studies would be of benefit to explore at the tumour level a possible activation of ftorafur into 5-FU via CYP2A6 and the possible modulation of this enzymatic pathway.

These possibilities to achieve pharmacomodulation of oral fluoropyrimidines, notably for UFT and capecitabine, opens up the prospect of establishing significant novel treatment protocols based on drug combinations. This increasing number of oral formulations for several cytotoxic drugs may well make it possible to provide ‘all oral’ treatments with advantages for patients in terms of quality of life.
